# Safety assessment of *Enterococcus lactis* based on comparative genomics and phenotypic analysis

**DOI:** 10.3389/fmicb.2023.1196558

**Published:** 2023-05-22

**Authors:** Jingda Lu, Tingting Shen, Yixin Zhang, Xinwei Ma, Sheng Xu, Sameh Awad, Muying Du, Zhi Zhong

**Affiliations:** ^1^Key Laboratory of Dairy Biotechnology and Engineering, Ministry of Education, Inner Mongolia Agricultural University, Hohhot, China; ^2^Key Laboratory of Dairy Products Processing, Ministry of Agriculture and Rural Affairs, Inner Mongolia Agricultural University, Hohhot, China; ^3^Inner Mongolia Key Laboratory of Dairy Biotechnology and Engineering, Hohhot, China; ^4^Department of Dairy Science and Technology, Faculty of Agriculture, Alexandria University, Alexandria, Egypt; ^5^College of Food Science, Southwest University, Chongqing, China; ^6^Chinese-Hungarian Cooperative Research Centre for Food Science, Southwest University, Chongqing, China

**Keywords:** *Enterococcus lactis*, antibiotic resistance, comparative genomics analysis, genome-wide association study, safety assessment

## Abstract

*Enterococcus faecium* is sometimes used in food production; however, its acquisition of antibiotic resistance has become an alarming health concern. The *E. lactis* species is closely related to *E. faecium* and has good probiotic potential. This study aimed to investigate the antibiotic resistance of *E. lactis.* We analyzed the antibiotic resistance phenotype and whole-genome sequences of 60 *E. lactis* isolates (23, 29, and 8 isolates from dairy products, Rice wine Koji, and human feces, respectively). These isolates showed varying degree of resistance to 13 antibiotics, and were sensitive to ampicillin and linezolid. The *E. lactis* genomes carried only a subset of commonly reported antibiotic resistance genes (ARGs) in *E. faecium*. Five ARGs were detected across the investigated *E. lactis*, including two universally present genes (*msrC* and *AAC(6′)-Ii*) and three rarely detected ARGs (*tet(L)*, *tetM*, and *efmA*). To identify other undescribed antibiotic resistance-encoding genes, a genome-wide association study was performed, returning 160 potential resistance genes that were associated with six antibiotics, namely chloramphenicol, vancomycin, clindamycin, erythromycin, quinupristin-dalfopristin, and rifampicin. Only around one-third of these genes encode known biological functions, including cellular metabolism, membrane transport, and DNA synthesis. This work identified interesting targets for future study of antibiotic resistance in *E. lactis*. The fact that the lower number of ARGs present in *E. lactis* supports that it may be an alternative to *E. faecalis* for use in the food industry. Data generated in this work is of interest to the dairy industry.

## 1. Introduction

*Enterococcus* are lactic acid bacteria. They are widely distributed in natural environments and are common gut commensals of humans and animals. Members of this bacterial family are acid- and heat-resistant (García-Solache and Rice, 2019), and with strong intestinal adhesion ability (Cebrián et al., 2012). However, this genus is also considered conditional pathogens, as they occasionally cause clinical diseases, such as bacteremia, infective endocarditis, urinary tract infection and so on (Gao et al., 2018). Moreover, the emergence of vancomycin-resistant *enterococci* (VRE) and the subsequent challenge in clinical treatment of VRE have received much attention in recent years (Eisenberger et al., 2020).

The taxonomic classification of the *Enterococcus* genus has been continuously changing since the last century. *Enterococcus* was originally a subspecies under the *Streptococcus* genus (Andrewes and Horder, 1906), and this group of bacteria were assigned to an independent genus in 1903 (Franz et al., 1999). *Enterococcus faecium* and *E. faecalis* are the two largest species under this genus, and they have been classified into different species in 1984 (Schleifer and Kilpper-Bälz, 1984). They are also the most characterized common species within the *Enterococcus* genus. The *E. lactis* was firstly isolated from dairy products in 2012 (Morandi et al., 2012), which was considered closely related to *E. faecium*. However, differences in the 16S rRNA gene sequence (Sukhodolets et al., 2005) and carbohydrate metabolism (Morandi et al., 2011) support that *E. lactis* should be assigned to an independent species from *E. faecium*.

Although *E. faecium* is widely used in the field of food and even as probiotics (De Vuyst and Vandamme, 1994; Moreno et al., 2006), the clinical infection rate caused by this bacterial species has increased sharply, and the discussion on whether *E. faecium* can be used as probiotics has become more and more intense. Compared with *E. faecium*, *E. lactis* lacks specific virulence and antibiotic resistance genes (ARGs), which make *E. lactis* a better choice to be used in the food and healthy food industries compared with *E. faecium* (Kim et al., 2022). However, a comprehensive food safety assessment of *E. lactis* is still lacking. Given the overall medical concern of the *enterococci* genus, it is necessary to investigate the antibiotic resistance in *E. lactis*.

Therefore, in this study, we comparatively analyzed the antibiotic-resistant phenotype (based on 15 antibiotics) and genomics of 60 *E. lactis* strains isolated from food (dairy product and Rice wine Koji) and human feces. By using a genome-wide association study (GWAS), we identified potential ARGs in the *E. lactis* genomes. This study has provided a preliminary evaluation of the antibiotic resistance of *E. lactis* in dairy products, Rice wine Koji, and human feces.

## 2. Materials and methods

### 2.1. Bacterial isolates and genomes

A total of 60 *E. lactis* isolates were analyzed in this study (Supplementary Table S1). Our laboratory isolated the bacteria from natural fermented dairy products (23 isolates), Rice wine Koji (29 isolates), and human feces (8 isolates) during 2007–2016. Nine of these isolates were previously sequenced (Zhong et al., 2019). In this study, 51 novel *E. lactis* genomes were sequenced.

### 2.2. *E. lactis* antibiotic susceptibility test

We determined the antibiotic resistance profile of the isolated *E. lactis* to 15 commonly used antibiotics, including ampicillin, vancomycin, gentamicin, kanamycin, streptomycin, erythromycin, clindamycin, tetracycline, chloramphenicol, quinupritin-dalfopristin, linezolid, ciprofloxacin, rifampicin, neomycin, and trimethoprim, by a standard microbroth dilution method (Cockerill et al., 2012; EFSA Panel on Additives and Products or Substances Used in Animal Feed (FEEDAP), 2012). The results were expressed in minimum inhibitory concentration (MIC).

### 2.3. DNA extraction

Each clonal isolate was aerobically cultured and subcultured in de Man, Rogosa and Sharpe liquid medium at 37°C for 24 h. Each bacterial subculture was checked for purity by microscopic examination. The genomic DNA of pure bacterial subculture of each isolate was extracted by using the Omega Biotek E.Z.N.A. Bacteria DNA Mini Kit (D3350-02, Omega Bio-tek, Inc., Norcross, GA, United States), and the purity and concentration of DNA were assured by using an ultraviolet spectrophotometer (NanoDrop ND-1000, Thermo Fisher Scientific Inc., Wilmington, DE, United States). Samples of DNA meeting the quality requirements (OD260/280 = 1.8 to 2.0, >6 μg) were kept frozen in a refrigerator (−20°C) for whole-genome sequencing.

### 2.4. Whole-genome sequencing, assembly, and annotation

The whole-genome sequencing of sequencing of *enterococcus* was done using the Illumina HiSeq platform (Illumina Inc., United States) by generating 2 × 150 bp paired-end libraries using the Nextera DNA Sample Preparation Kit (Illumina Inc., United States) following the manufacturer’s instructions. Then, the whole-genome sequencing was conducted on an Illumina HiSeq sequencing platform (Illumina Inc. United States; Lepage et al., 2006). The high quality pair-end reads data were first assembled using SOAP denovo v1.06 (Luo et al., 2012), and the genome gaps were filled with the GapCloser.^1^

### 2.5. Calculation of average nucleotide identity

The ANI analysis examined the intra-species relationship based on genome sequence similarity. The analysis was performed by the method described in Goris et al. (2007) with a cut-off level was 95%.

### 2.6. Construction of core- and pan-genomes

The coding sequences of 60 *E. lactis* isolates were predicted and annotated by Prokka v1.11 software (Seemann, 2014). Roary v3.6.1 software (Page et al., 2015) was used to identify the core- and pan-gene sets of *E. lactis*. The same gene family was defined based on amino acid similarity of encoding sequences (>95%). The core-gene set was constructed by identifying gene families shared across all isolates, while all identified gene families were used to construct the pan-gene set. Finally, the PanGP software^2^ was used to construct the pan-/core-gene accumulation curves.

### 2.7. Construction of the phylogenetic tree

A core-gene phylogenetic tree was constructed by based on the core-gene nucleotide sequences using the Neighbor-Joining (NJ) method with TreeBeST software (Vilella et al., 2009). The tree was visualized using the online software iTol^3^ (Letunic and Bork, 2019), with the Bootstrap parameter set to 1,000.

### 2.8. Identification of ARGs

To uncover ARGs in the 60 *E. lactis* isolates, the identified protein sequences from these genomes were BLASTp-compared against the Comprehensive Antibiotic Resistance Database (CARD; http://arpcard.mcmaster.ca), with a cut-off level of similarity of >85%, *E* values < le-15 (Tang et al., 2019).

### 2.9. Identification of virulence factors

To uncover virulence factors in the 60 *E. lactis* isolates, the identified protein sequences of these genomes were compared against the Virulence Factor Database,^4^ with a cut-off level of *E*-value <1e-15 and sequence identity >95%.

### 2.10. Identification of potential ARGs by GWAS

A GWAS was performed to identify potential ARGs using Scoary software (Brynildsrud et al., 2016). The MIC results of 60 *E. lactis* isolates were used as phenotypic characteristics. The screening criteria for potential ARGs were naive *p* < 0.05 and empirical *p* < 0.05. The false discovery rate (corrected by Benjamini and Hochberg method) < 0.05 was considered statistically significance. Identified putative ARGs were annotated through the Kyoto Encyclopedia of Genes and Genomes database.

### 2.11. Statistical analysis

The ggpubr package in R (V4.1.2) was used to draw the boxplots. TBtools (Chen et al., 2020) was used to draw the heatmap. Data analyses were performed using SPSS 26.

### 2.12. Data availability statement

The genomic sequences of the nine previously sequenced *E. lactis* were retrieved from DDBJ/ENA/GenBank database under the accession number PGPI00000000 to PGPM00000000. Currently assembled genomes of the other 51 isolates were deposited in DDBJ/ENA/GenBank database under the accession number JAIZWO000000000 to JAIZYM000000000 (Supplementary Table S1).

## 3. Results

### 3.1. Antibiotic-resistant phenotype of *Enterococcus lactis* isolated from dairy products

**Figure 1:**
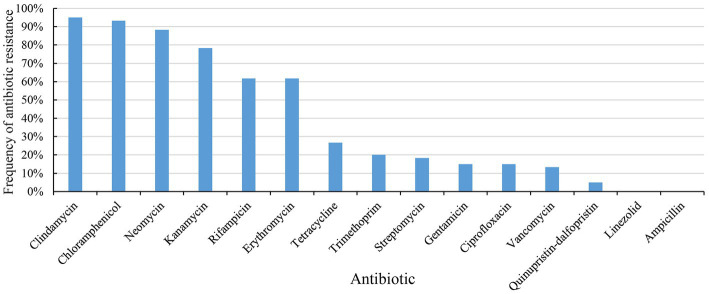
Antibiotic resistance of 60 strains of *Enterococcus lactis* to 15 antibiotics.

60 strains of *E. lactis* were tested for resistance to 15 common antibiotics by a microbroth dilution method (Supplementary Table S2). The species showed the strongest resistance to clindamycin (57/60, 95%), followed by chloramphenicol (56/60, 93%). The resistance rate of *E. lactis* to neomycin (53/60, 88%), kanamycin (47/60, 78%), rifampicin (37/60, 62%), and erythromycin (37/60, 62%) were higher than 50%, and the species had a low resistance rate (<50%) to tetracycline (16/60, 27%), trimethoprim (12/60, 20%), streptomycin (11/60, 18%), gentamicin (9/60, 15%), ciprofloxacin (9/60, 15%), vancomycin (8/60, 13%), and quinupristin-dalfopristin (3/60, 5%). All tested isolates were sensitive to linezolid and ampicillin (Figure 1).

The phenotype of antibiotics resistance of *E. lactis* varied greatly. 60 *E. lactis* isolates were resistant to 3 to 12 different antibiotics, and 48 isolates were resistant to 5 or more antibiotics. Around half of the studied isolates were originated from Rice wine Koji, and three isolates showed a broad spectrum resistance to more than 10 different antibiotics (20-3, 15-3, and 19-3, resistant to 12, 11, and 10 different antibiotics, respectively). The isolates dairy-associated isolates, XJ28301 and XJ28304, showed the narrowest antibiotic resistance spectrum.

### 3.2. General characteristics of the *Enterococcus lactis* genomes

**Figure 2:**
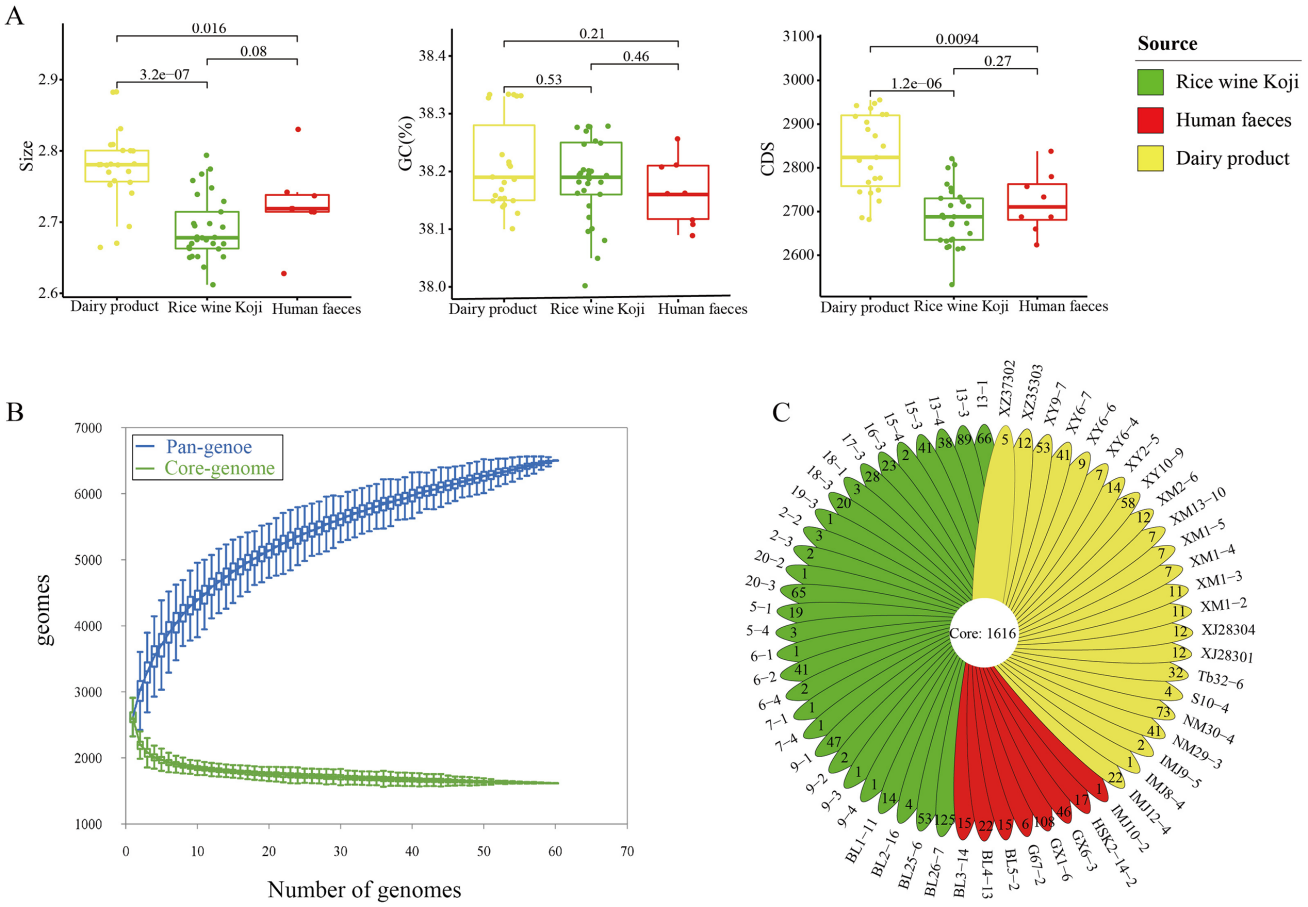
Genome characteristics of the studied *Enterococcus lactis*. **(A)** Mean genome size, G + C content, and number of coding sequences in dairy, Rice wine Koji, and human fecal isolates. Significant differences between sample pairs were evaluated with wilcox test, and the generated *p* values are shown. **(B)** Pan- and core-genomes of 60 strains of *Enterococcus lactis*. Upper and lower whiskers represent pan-and core-genomes. **(C)** Isolate-specific genes of the studied *Enterococcus lactis* genomes.

Comparative genomics analysis was performed on the 60 *E. lactis* isolates. Their average genome size was 2.73 ± 0.06 Mb (range = 2.61–2.88 Mb), and the average number of predicted genes was 2,748 ± 100. The overall G + C content of *E. lactis* was low, ranging from 38.00 to 38.33%. The dairy isolates had a significant larger genome size and a higher number of coding sequences compared with isolates from Rice wine Koji and human feces (*p* < 0.05), but no significant difference was observed in the GC content among isolates of three sources (Figure 2A).

### 3.3. The core- and pan-genomes of *Enterococcus lactis*

Then, we determined the core- and pan-gene sets of the investigated isolates. The core gene set comprised 1,616 gene families, while the pan-gene set comprised 6,502 gene families (Figure 2B). The core genes accounted for 58.80% of the total number of predicted genes (2,748 genes). In other words, nearly 41.20% of the predicted genes in each genome were accessory genes. The pan-gene accumulation curve did not reach a plateau, suggesting an open pan-genomes and more accessory genes would be identified as more genomes are added into the dataset (Figure 2B).

The 60 *E. lactis* isolates contained an average of 24.51 isolate-specific genes. The human fecal isolates (28.75 genes) had a significantly higher average number of isolate-specific genes compared with isolates from Rice wine Koji and dairy products (25.11 and 19.69, respectively; *p* < 0.05; Figure 2C). Average nucleotide identity of *E. lactis*.

**Figure 3:**
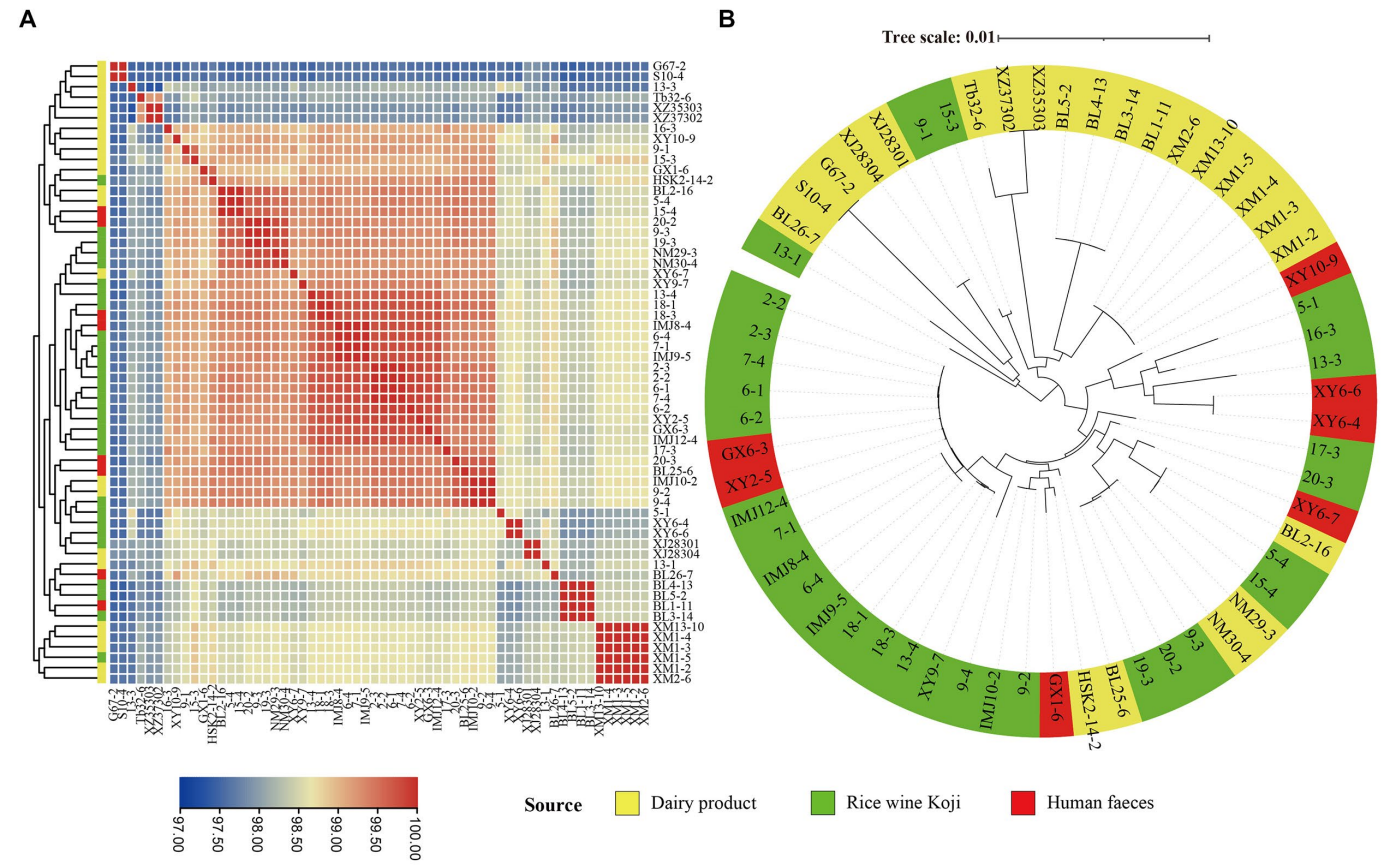
Intra-species genomic similarity. The analyses were performed based on the genomes of 60 *Enterococcus lactis* isolates. **(A)** Heatmap showing average nucleotide identity (ANI). The color scale represents ANI level; and a darker red indicates a higher similarity, while a darker blue indicates a lower similarity. **(B)** Core-gene phylogenetic tree of the *E. lactis* isolates. The phylogenetic tree was constructed using the DNA sequences of 1,616 core genes from these isolates.

In order to evaluate the intraspecific genome sequence similarity, the ANI values were calculated and were shown in a heatmap (Figure 3A). All the pairwise ANI values were over 95% (range = 97.32 to 99.99%), suggesting all of the investigated isolates belonged to the *E. lactis* species (Goris et al., 2007).

### 3.4. Phylogenetic reconstruction of *Enterococcus lactis*

In order to study the evolutionary relationships of *E. lactis* from different isolation sources, a phylogenetic tree was constructed based on 1,616 core gene nucleotide sequence (Figure 3B). Two major clusters were formed, representing mainly isolates from Rice wine Koji and human feces (the larger cluster) and isolates from dairy products (the smaller cluster), although there were few exceptions. Three isolates from Rice wine Koji (13-1, 9-1, 15-3) clustered were distributed in the cluster of dairy isolates, and five isolates from dairy products (BL2-16, NM29-3, NM30-4, BL25-6, HSK2-14-2) were distributed in the Rice wine Koji isolate cluster. These results suggested that the dairy isolates diverge from those originated from Rice wine Koji and human feces.

### 3.5. Virulence factors in *Enterococcus lactis* genomes

**Figure 4:**
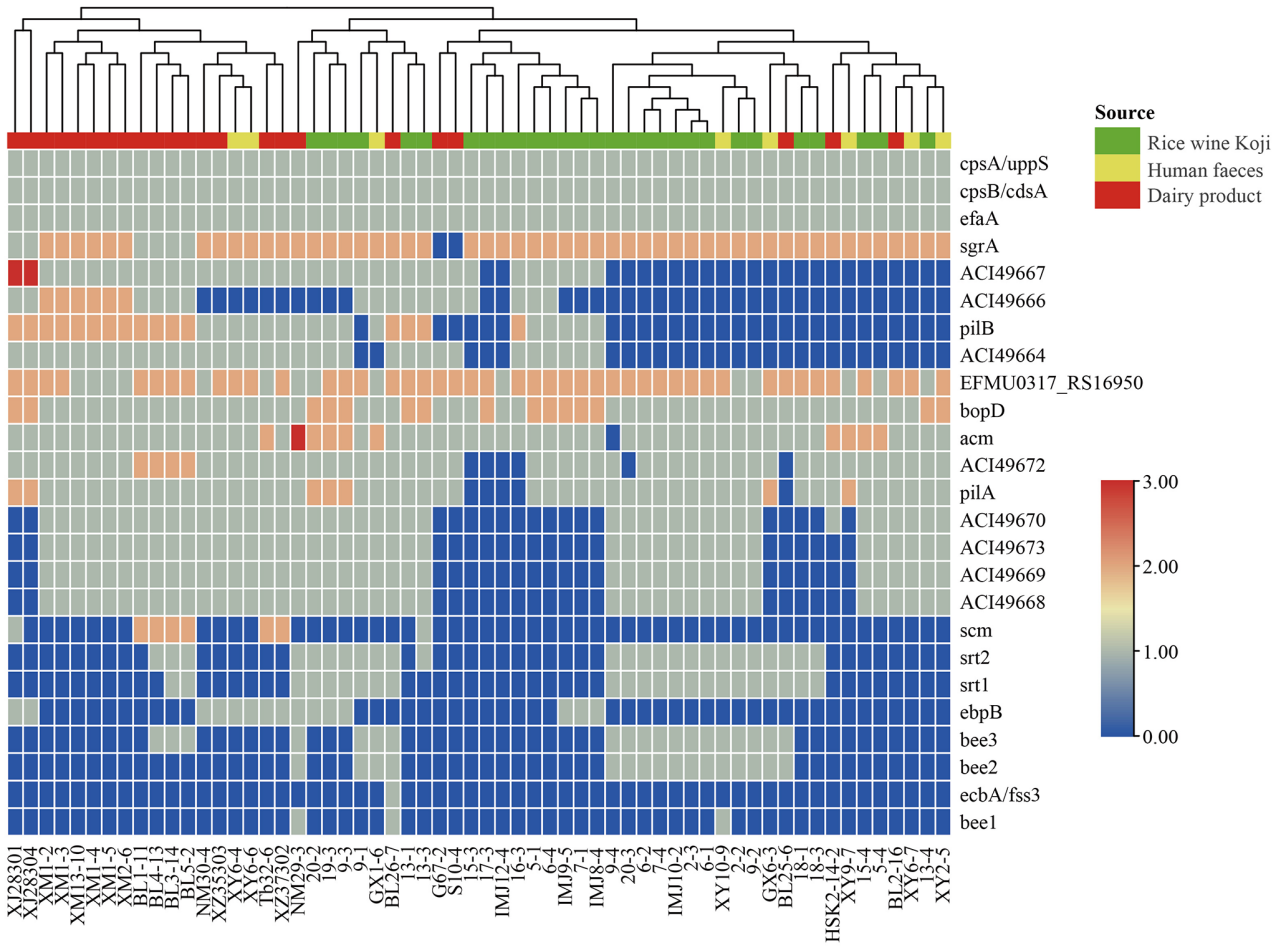
Cluster analysis based on the profile of virulence factors identified in 60 *Enterococcus lactis* genomes. The color scale indicates the number of virulence factors.

To identify potential virulence factors in the *E. lactis* genomes, the coding sequences of 60 *E. lactis* isolates were compared against the Virulence Factor Database. A total of 25 virulence factors were detected, which were mainly related to adhesion, immune regulation, and biofilm formation; and the category of adhesion-related virulence factors had the high number of virulence factors, namely *cps*A/uppS, cpsB/cdsA, *efa*A, *EFMU*0317_RS16950, *bop*D, and *acm* (Figure 4).

### 3.6. Antibiotic resistance genes in *Enterococcus lactis* genomes

**Figure 5:**
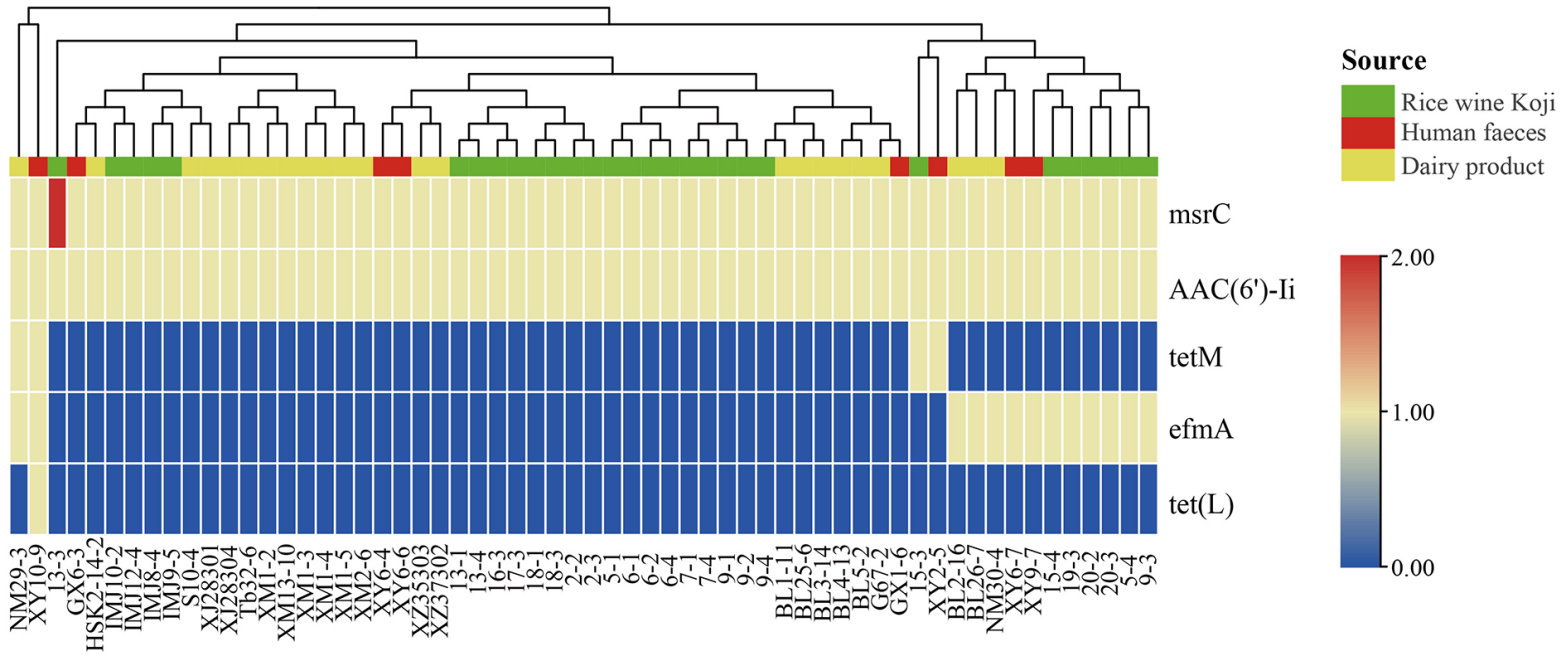
Cluster analysis based on the profile of antibiotic resistance genes identified in 60 *Enterococcus lactis* genomes. The color scale indicates the number of antibiotic resistance genes.

To identify known ARGs in the *E. lactis* genomes, the coding sequences of 60 *E. lactis* isolates were BLAST-search across the CARD database, returning a total of five ARGs (Figure 5). Two of these ARGs, *msrC* and *AAC(6′)-Ii*, were universal across all isolates. *TetM* and *efmA* were present in all three isolation sources. *TetL* was detected only in a fecal isolate XY10-9. The human fecal isolate (XY10-9) and the dairy isolate (NM29-3) contained the highest numbers of ARGs, five and four ARGs, respectively (Figure 5).

### 3.7. Identification of potential antibiotic-resistant phenotype-encoding genes by GWAS

To identify potential antibiotic-resistant phenotype-encoding genes other than the known ARGs, a GWAS was performed using Scoary software. Our analysis returned 160 potential antibiotic resistance-encoding genes that were associated with six antibiotics, including: chloramphenicol (123 associated genes; only 40 encode known function proteins), vancomycin (15 associated genes; 10 encode known proteins), clindamycin (12 associated genes; only two encode known function proteins), erythromycin (5 associated genes; all annotated as hypothetical proteins), quinupristin-dalfopristin (3 associated genes; all annotated as hypothetical proteins), and rifampicin (2 associated genes; both encoding known function proteins). The identified genes were annotated by the Kyoto Encyclopedia of Genes and Genomes database gene annotation; 54 genes encoded proteins of known function, and the remaining 106 genes encoded hypothetical proteins (Supplementary Table S3).

## 4. Discussion

The *Enterococcus* genus is often found in fermented dairy products, and some of them have been used as fermented food starter and probiotics. Most previous research of *Enterococcus* focuses on their medical aspects, especially antibiotic resistance acquisition and genomics of clinically-associated strains, due to the fact that more than of nosocomial infections worldwide are related to this group of bacteria (Arias and Murray, 2012). However, in recent years, there has been a growing interest in studying food-originated *Enterococcus species* because of their increasing use in the food industry. *Enterococcus lactis* is a relatively new species and is closely related to *E. faecium*. This species is advantageous over *E. faecium* for use in the food industry because of its lack of obvious virulence and ARGs. Nevertheless, safety risk is a prime concern for any food use bacteria, and few reports have evaluated the risk of spread of antibiotic resistance in *E. lactis.* Thus, in this study, we determined the antibiotic resistance profile of 60 isolates, most of which were food-originated (from dairy products and Rice wine Koji). We identified potential virulence factors and known ARGs in their genomes, and implemented a GWAS approach to uncover potential antibiotic resistance-encoding genes.

We first determined the antibiotic susceptibility of the collected isolates to 15 commonly used antibiotics, which would provide an idea of the antibiotic resistance spectrum of food-originated isolates of this species and a starting point for safety assessement. Our results indicated that 95% of the investigated isolates were resistant to clindamycin, which is surprising. Intrinsic antibiotic resistance of *enterococci* to lincosamide antibiotics has been reported in multiple studies (Klare et al., 2003). 63% of our isolates were resistant to erythromycin, which corroborates the observation of a high prevalance of *enterococci* to erythromycin (Lopes et al., 2005). The major reason of resistance to macrolide antibiotics like erythromycin is horizontal gene transfer of drug-resistant gene elements through plasmids and transposons (Santagati et al., 2000; Arredondo-Alonso et al., 2020). On the other hand, linezolid is an important antibiotic for treating VRE infection, and all the investigated isolates were sensitive to linezolid, which is consistent with Kürekci et al. (2016). We also observed a relatively low prevalence of vancomycin resistance (13%). Glycopeptide antibiotics like vancomycin are the reserve antibiotics for resistant multidrug-resistant *Enterococcus species*. A high prevalence of vancomycin resisance (>50%) was previously reported in Turkish cheese-associted *enterococci* (Çitak et al., 2004). As antibiotic resistance became an alarming global health concern, the use of antibiotics in animal production was banned in 2006 (Demirgül and Tuncer, 2017). The implementation of such measure for over a decade has slowly brought down the antibiotic resistance in environmental *enterococci* isolates, and continuous monitoring of the spread of antibiotic resistance in clinical and environmental bacteria is crucial.

The overall ranges of genome size and GC content of *E. lactis* are similar to that of the genomes of *E. faecalis* and *E. faecium* (Sanderson et al., 2020). *Enterococci* carry a number of virulence factors in their genomes. For example, the *efaA* gene is a known virulent factor-encoding gene in *E. faecalis*, and the vast majority of *E. faecalis* food isolates was found to contain the *efaA* gene (Eaton and Gasson, 2001), which can be used as a gene target for molecular detection of *E. faecalis*. Our study consistently found that the *efaA* gene is present in all *E. lactis* isolates. Other commonly found virulence factors in *enterococci*, such as *Acm* and *bopD*, are also widely detected among the current *lactis* isolates. However, other high prevalent virulence factors in *enterococci*, such as gelE (responsible for producing extracellular enzymes; Bertelli et al., 2017) and *esp* (encoding surface proteins; Das et al., 2020) are not present in *E. lactis*. Instead, *E. lactis* possess two immune regulation and capsular polysaccharide production-associated virulence factors, cpsA/uppS (encoding an undecaprenyl diphosphate synthase) and cpsB/cdsA (encoding a phosphatidate cytidylyltransferase), which are less reported in *enterococci*.

Compared with *E. faecium* and *E. faecalis*, a relatively small number of ARGs were detected in *E. lactis*. These detected genes represent a subset of consistently reported ARGs in *E. faecium*. For example, the *msrC* is a chromosomal gene that encodes an ABC efflux pump, conferring resistance to macrolides and streptosporin class B antibiotics in *E. faecium* (Munita and Arias, 2016). *AAC(6′)-II* encodes an aminoglycoside acetyltransferase that confers resistance to several aminoglycoside antibiotics (Costa et al., 1993), and it is an important determinant of microbial resistance in *E. faecium* (Draker et al., 2003). *EfmA* encodes is mainly responsible for promoting the permeability enzyme of the major facilitator superfamily transporters, conferring fluoroquinolone and macrolide to *E. faecium* (Vasconcellos et al., 2022). TetM and tetL genes are ribosome protective proteins that promote tetracycline resistance (Connell et al., 2003). Neela et al. (2007) reported that although some strains carry the *tetM* gene, they are still sensitive to tetracycline, indicating that the detection frequency of tetM gene in the strain does not represent the resistance level. Therefore, it is still necessary to gene expression to determine the drug-resistant phenotype (Neela et al., 2007). It is worth noting that virulence determinants like the vancomycin ARG cluster was detected in the investigated *E. lactis*, suggesting that the ARG distribution in the *E. lactis* genomes differs from that of *E. faecium* and *E. faecalis*, making *E. lactis* a better choice for food use.

Finally, we performed a GWAS by applying the antibiotic resistance phenotype data and the whole-genome sequences of the *E. lactis* to locate yet unidentified antibiotic resistance-encoding genes based on association. A total of 160 genes were found to associate with six different antibiotics, although around two-thirds of which were annotated as hypothetical proteins (106/160, 66.3%). Most of the known function proteins are related to chloramphenicol resistance and vancomycin resistance. Interestingly, some of these genes encode function related to cellular metabolism, membrane transport, and DNA synthesis. For example, chloramphenicol resistance is associated with genes encoding lactose-inducible lactose-phosphotransferase operon (*lacA* to *lacG*; van Rooijen et al., 1991) and the mannose PTS system (*manXa*, *manY*, *manZ*), while vancomycin resistance is associated with genes encoding an ABC transport system ATP-binding protein (*yknY*), an ABC transport system permease protein (*macB*), and several putative proteins related with ribonucleotide reductases (*nrdE*, *nrdF*, *nrdI*). It is interesting to note that ribonucleotide reductase is an essential enzyme for *de novo* synthesis of DNA building; it has been proposed as antimicrobial drug target towards opportunistic pathogens like *Pseudomonas aeruginosa* (Tholander and Sjöberg, 2012).

One limitation of this study is that it remains at genomic-level analysis, so the functional roles of these known function proteins and hypothetical proteins in antibiotic resistance are not clear. Nevertheless, this study has provided interesting targets for future study aiming to comprehensively elucidate the antibiotic resistance in *E. lactis*.

## 5. Conclusion

In conclusion, this study comprehensively analyzed the antibiotic resistance in *E. lactis*. We identified some putative virulence factors and ARGs in the investigated *E. lactis* genomes, but some virulence determinants (such as vancomycin ARG cluster) commonly present in other *enterococal* species of clinical concern have not detected. Then, by conducting a GWAS, a number of potential antibiotic resistance-encoding genes have also been found, which are interesting targets for future study of antibiotic resistance in *E. lactis*. Moreover, the fact that the lower number of ARGs present in *E. lactis* supports that it may be an alternative to *E. faecalis* for use in the food industry.

Further studies should expand the number of isolates, so as to increase the confidence of results generated by GWAS. Nevertheless, this study has provided a starting point for studying the antibiotic resistance in a potential food use species, *E. lactis*.

## Data availability statement

The datasets presented in this study can be found in online repositories. The names of the repository/repositories and accession number(s) can be found in the article/Supplementary material.

## Author contributions

JL and ZZ designed the study. ZZ, MD, and JL performed the de novo assembly and comparative genomics analyses. JL, TS, XM, YZ, and SX analyzed the antibiotic resistance profile of *E. lactis* isolates. TS, ZZ, and SA wrote the manuscript. All authors contributed to the article and approved the submitted version.

## Funding

The study was supported by the Science and Technology Project of College of Food Science and Engineering, Inner Mongolia Agricultural University (SPKJ201905) and National key research and development program from Ministry of Science and Technology, China (no. 2022YFE0120800).

## Conflict of interest

The authors declare that the research was conducted in the absence of any commercial or financial relationships that could be construed as a potential conflict of interest.

## Publisher’s note

All claims expressed in this article are solely those of the authors and do not necessarily represent those of their affiliated organizations, or those of the publisher, the editors and the reviewers. Any product that may be evaluated in this article, or claim that may be made by its manufacturer, is not guaranteed or endorsed by the publisher.

## Footnotes

^1^http://sourceforgE.net/projects/soapdenovo2/files/GapCloser/

^2^https://pangp.zhaopage.com/

^3^https://itol.embl.de/

^4^http://www.mgc.ac.cn/VFs/main.htm
